# The Role of IL-6, 8, and 10, sTNFr, CRP, and Pancreatic Elastase in the Prediction of Systemic Complications in Patients with Acute Pancreatitis

**DOI:** 10.1155/2013/282645

**Published:** 2013-02-12

**Authors:** E. Fisic, G. Poropat, L. Bilic-Zulle, V. Licul, S. Milic, D. Stimac

**Affiliations:** ^1^Department of Laboratory Diagnostics, University Hospital Rijeka, Kresimirova 42, 51000 Rijeka, Croatia; ^2^Division of Gastroenterology, Department of Internal Medicine, University Hospital Rijeka, Kresimirova 42, 51000 Rijeka, Croatia

## Abstract

*Background and Aim*. Early assessment of severity in acute pancreatitis (AP) is a key measure to provide rational and effective management. The aim of our study is to determine the prognostic value of interleukins (IL) 6, 8, and 10, soluble receptor for tumor necrosis factor (sTNFr), pancreatic elastase (E1), and C-reactive protein (CRP) as predictors of systemic complications in AP. *Patients and Methods*. A hundred and fifty patients with confirmed AP were enrolled in the study. The severity of AP was defined according to Atlanta criteria. Measurements of interleukins and sTNFr were performed on the first day of admission. CRP and E1 levels were assessed on admission and after 48 hours. ROC analysis was performed for all parameters. *Results*. Interleukins and sTNFr significantly differentiated patients with systemic complications from those without. Elevation of IL-6 showed the highest significance as a predictor (*P* = 0.001). CRP and elastase levels did not differ between mild and severe cases on admission, but reached statistical significance when measured on the third day (*P* = 0.002 and *P* = 0.001, resp.). *Conclusion*. Our study confirmed that IL-6, IL-8, IL-10, and sTNFr measured on admission, and CRP and pancreatic elastase measured on third day of admission represent valuable prognostic factors of severity and systemic complications of AP.

## 1. Background and Aim

Acute pancreatitis (AP) is a common and potentially lethal acute inflammatory disease with an estimated overall mortality rate of 2% to 5%, and a significant burden of morbidity and health care costs [1]. Although usually self-limiting, up to 20% of patients develop a severe form of disease, which can lead to a systemic inflammatory response and multiple organ dysfunction and failure [2]. The two prevailing causes of AP are excessive alcohol consumption, most common in men, and gallstones, most common in women, which seem to act through different pathogenic mechanisms to induce pancreatic acinar cell damage. Several multifactorial scoring systems and routine clinical and biochemical parameters measured on admission and during the first 48 hours of hospitalization are used to estimate severity and promptly provide a rational and effective management.

Systemic manifestations of a disease initially limited to the pancreas are thought to be mediated by a variety of pro- and anti-inflammatory mediators released from the pancreas and various other sources during the course of the disease. Several cytokines play a crucial role in the pathogenesis of AP by driving the additional inflammatory response which leads to tissue damage and organ dysfunction. Local recruitment and activation of inflammatory cells in AP may lead to the production of proinflammatory cytokines, such as interleukins (IL) 6, 8, and tumor necrosis factor alpha (TNF-alpha) or his soluble receptor (sTNFr), as well as anti-inflammatory IL-10. These mediators have been mostly studied as markers of severity of acute pancreatitis. Another commonly applied and one of the first used markers for this purpose was C-reactive protein (CRP). Different studies showed that a CRP value over 200 mg/L obtained at 48 hours after onset of symptoms is highly predictive of pancreatic necrosis [3]. The use of pancreatic elastase for the differentiation between mild and severe cases of AP has also been investigated, yielding however conflicting results [4]. 

Still, insufficiently is known of the relationship between the clinical course of AP in humans and the dynamic of the major cytokines, in the presence or absence of pancreatic necrosis and distant organ complications. The purpose of our study was to determine the potential clinical value of interleukins (IL-6, IL-8, IL-10), soluble receptor for tumor necrosis factor (sTNFr), pancreatic elastase, and C-reactive protein as biochemical markers for predicting development of systemic complications in patients with AP. 

## 2. Patients and Methods

### 2.1. Patients

A hundred and fifty patients with acute pancreatitis were prospectively entered into the study during a two-year period. The diagnosis of AP was made on the basis of a consistent clinical picture combined with a 3-fold increase of serum amylase or a 3-fold increase of serum lipase, and consistent morphological findings obtained by an ultrasound scan and/or computed tomography scan within the first 72 hours of admission. The severity of AP was assessed according to the Atlanta classification. All patients irrespective of disease severity were included in the study. Mild acute pancreatitis (MAP) was defined as confirmed AP without signs of major complications, while severe acute pancreatitis (SAP) was associated with the development of one or more local or systemic complications. Local complications included pancreatic tissue necrosis, as well as the formation of acute fluid collections, pancreatic pseudocyst, and abscess. Systemic complications assumed the presence of persistent systemic inflammatory response syndrome (SIRS) and/or developing organ failure. SIRS was defined by 2 or more of the following criteria for >48 hours: heart rate >90 beats/min; rectal temperature <36°C or >38°C; white blood count <4000 or >12,000 per mm^3^; and respirations >20/min or pCO_2_ < 32 mmHg. Organ failure was defined as shock (systolic blood pressure <90 mmHg), pulmonary insufficiency (pO_2_ < 60 mmHg), renal failure (creatinine >2 mg/dL, despite rehydration), and gastrointestinal bleeding (>500 mL/24 hours) [5, 6]. The study was performed according to local ethics committee regulations.

### 2.2. Methods

Blood samples from patients were obtained on admission and after 48 hours. Samples for IL-6, IL-8, IL-10, IL-15, IL-17, and sTNFr were aliquoted in portions and stored at −20°C, not longer than two months. Measurements were performed using a commercially available ELISA kit (R&D Systems Inc., Minneapolis, USA) on a standard ELISA reader according to the manufacturer's instructions. Pancreatic elastase was analysed using a commercially available ELISA kit (ScheBo-Biotech, Giessen, Germany). Levels of CRP and other routine laboratory assessments were completed on biochemistry analyser Olympus AU 640 (Mishima Olympus, Japan). Measurements of interleukins and sTNFr were performed on samples obtained on the first day of admission. CRP and pancreatic elastase levels were assessed on admission and after 48 hours.

### 2.3. Statistics

All variables are expressed as medians with 95% cofidence intervals (95% CI). Mann Whitney *U* test was used for comparison of independent samples. For differences between values of same parameters obtained on admission and after 48 hours Wilcoxon pair test for dependent samples was used. Receiver operating characteristic (ROC) curves and respective areas under curve (AUC) were established for biochemical prognostic factors. Cut-off values were chosen as values that achieved the highest sensitivity and specificity, as well as positive (PPV) and negative predictive values (NPVs). The proportion of patients without systemic complications was used as a measure of prevalence in performing ROC analysis. A value of *P* < 0.05 was considered statistically significant.

## 3. Results

A total of 150 patients with AP (71 male; median age 63; range 20–91) were included in the study. AP was considered severe in 28 patients (19%) and mild in 122 patients (81%). The etiology of AP was biliary in 68 patients (45%), alcoholic in 51 patients (34%), and other possible causes (hypertriglyceridemia, post-ERCP, idiopathic, etc.) in 31 patients (21%). 

The average value of IL-6 measured in the group of patients was higher than the upper limit of reference range recommended by the manufacturer (29 versus 12.5 pg/mL, resp.), whereas average values of other measured cytokines were within normal ranges. CRP measured on the first and third day of admission was above the upper limit of normal, as well as the average value of pancreatic elastase measured on the first day. Average values of pancreatic elastase measured on the third day were within the boundaries of recommended values (Table 1).

In the assessment of disease severity, average values of CRP and pancreatic elastase differed significantly between the first and third day of hospitalization, with a significant increase in CRP values and a significant decrease in serum concentrations of pancreatic elastase (Table 2). 

The comparison of the analyzed biochemical prognostic factors between acute pancreatitis patients who developed systemic complications and those who did not is shown in Table 3. We found a significant difference between the values of IL-6, IL-8, IL-10, and sTNFr evaluated on the first day of admission, and a significant difference between CRP and elastase values analyzed from samples taken on the third day. No significant difference was noted in the values of CRP and elastase on the first day between these two groups of patients. 

The effectiveness of the investigated biochemical parameters in the early recognition of patients with and without systemic complications was assessed using ROC analysis. Considering the area under the ROC curve, values of cytokines measured on the first day were statistically significant indicators for development of systemic complications. CRP and pancreatic elastase measured on the third day also reached statistical significance. Results from analysis are shown in Table 4.

**Figure 1:**
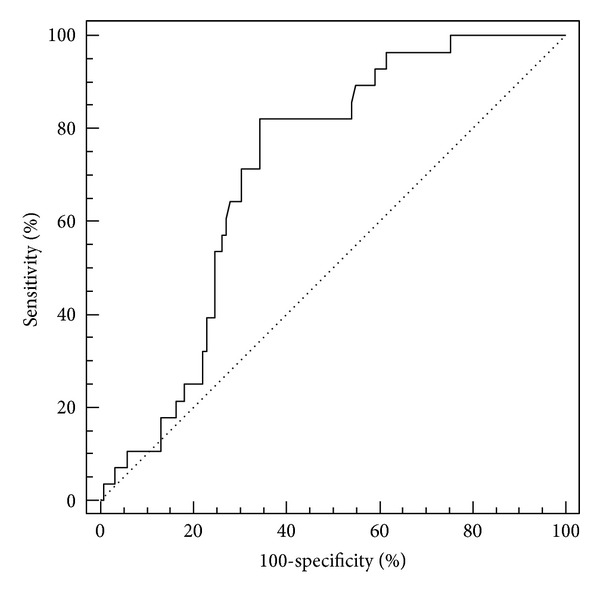
ROC curve for IL-6. Area under the curve (AUC) = 0.71; the limit value is 37.9 pg/mL.

**Figure 2:**
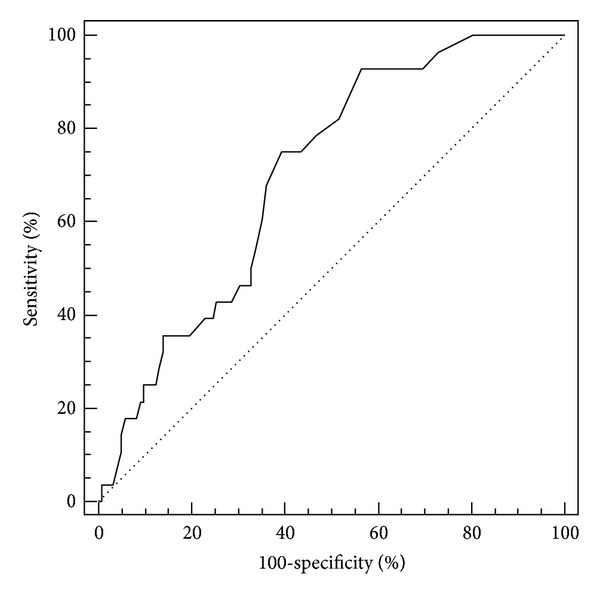
ROC curve for elastase measured on the third day of admission. Area under the curve (AUC) = 0.70; the limit value is 1.5 ng/mL.

**Figure 3:**
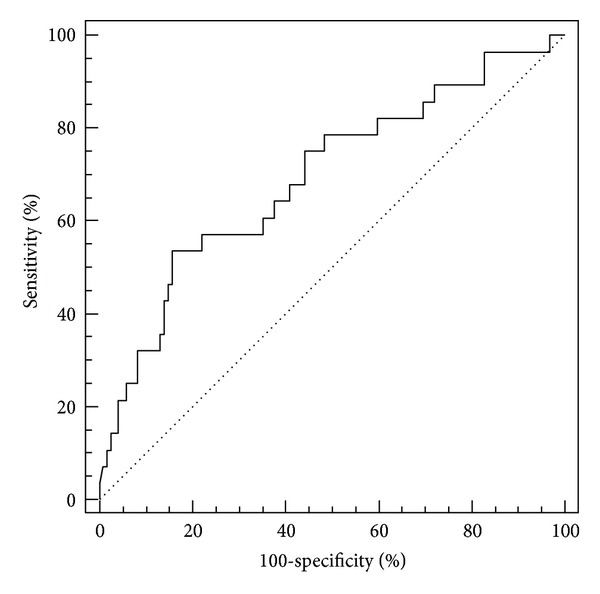
ROC curve for CRP measured on the third day of admission. Area under the curve (AUC) = 0.69; the limit value is 214 mg/L.

The largest area under the curve was for IL-6 (AUC = 0.71) and elastase on the third day (AUC = 0.70) (Figures 1 and 2). Elastase had a fairly high sensitivity of 92%, but a rather low specificity of 43%. The highest specificity (84%) was calculated for the marginal value of CRP measured on the third day, with a sensitivity of 54% (Figure 3). CRP and elastase measured on the first day had the lowest predictive value (AUC = 0.51 and 0.56, resp.) not reaching statistical significance. 

## 4. Discussion

In this study we examined the value of IL-6, IL-8, IL-10, sTNFr, CRP, and pancreatic elastase as predictors of systemic complications in AP. The need for an early risk recognition and determination of best possible treatment modalities led to a series of investigations trying to establish an objective, rational, and clinically manageable severity assessment tool in patients with AP. 

 The initial acinar cell damage in the early stage of acute pancreatitis of any etiology is caused by a hypersecretion of pancreatic proteolytic enzymes. As a result there is an overproduction of inflammatory mediators and free oxygen radicals. Tissue macrophages are the main source of proinflammatory and anti-inflammatory cytokines that attract neutrophils and more macrophages, and induce the production of proteases, elastases, and phospholipases. These enzymes, as well as free oxygen radicals cause tissue damage, mainly vascular endothelial necrosis which leads to circulatory stasis. The increase of proinflammatory and decrease of anti-inflammatory cytokines are crucial factors in the progression of inflammation of severe acute pancreatitis. The largest studies have focused on the role of TNF-alpha, IL-1, IL-6, and IL-10. Most of these studies have shown that the levels of proinflammatory cytokines (TNF-alpha, IL-1, IL-6) are higher in severe forms of AP, while levels of IL-10, which is anti-inflammatory agent are higher in patients with mild disease. The systemic manifestations of severe acute pancreatitis are not only caused by local inflammatory processes, but also by an excessive production and systemic spreading of inflammatory mediators. 

 We performed data analysis by dividing our patients into two groups. In one group we had patients who developed systemic complications (*N* = 28), and in the other those who had none (*N* = 122). The proportion of patients with systemic complications in our study correlates with the published data [7]. Our results show that the average value of IL-6 in patients with AP was above the upper limit of reference range recommended by the manufacturer, while average levels in controls were within normal ranges. ROC analysis was performed to evaluate the prognostic value of IL-6 to distinguish patients with systemic complications from those without, showing that patients with IL-6 concentrations greater than 37.9 pg/mL can be considered high risk in terms of developing systemic complications. We found a sensitivity of 82%, and a specificity of 65%, with a PPV of 35%, and an NPV of 94%. In our previous study, results differed slightly with a sensitivity and specificity of 68.7% and 69.9%, respectively, and PPV of 50%, and NPV of 83.6% [8]. These differences probably derive from the quality of available tests. Pezzilli et al. showed in their paper an AUC of 0.91, a sensitivity of 100%, and specificity of 83% [9]. These values in combination with serum lipase levels achieved a diagnostic and prognostic accuracy of 94%. The most likely cause is a smaller number of patients and the use of different tests for cytokine analysis. In another study, Jiang et al. determined the concentration of IL-6, TNF-alpha, and CRP over several days after admission and found that IL-6 has the highest sensitivity and specificity (100% and 89.7%, resp.) on the first day of admission [10]. Chen et al. analyzed, among other things, levels of IL-6, IL-8, and IL-10 in 78 patients undergoing endoscopic retrograde cholangiopancreatography (ERCP) and found that patients who suffered from post-ERCP pancreatitis have significantly higher concentrations of these cytokines [11]. Using a cut-off level of 36 pg/mL they found that the sensitivity and specificity for recognition of post-ERCP pancreatitis were 100% and 87%, respectively. 

 The role of the proinflammatory IL-8 in prediction of severity of acute pancreatitis seems less valuable than IL-6. Although it reached statistical significance (*P* < 0.008) in the differentiation of mild and severe disease forms at a threshold value of 42.5 pg/mL, it achieved a modest sensitivity and specificity of 68% and 67%, respectively. Same conclusions were obtained in previous studies by Pooran et al., and Berney et al. [12, 13].

Interleukin 10 has an anti-inflammatory role inhibiting the synthesis and release of other proinflammatory cytokines and free oxygen radicals from macrophages and T-helper lymphocytes. On the other hand, it shows a positive effect on the proliferation and differentiation of B lymphocytes promoting the production of immunoglobulins. Our results show that a limit for IL-10 that can be said to separate milder from more severe forms of AP is 7.2 pg/mL, with a sensitivity of 75%, and a specificity of 56%. Our previous study showed a lower sensitivity, and same specificity for a higher cut-off value (37 pg/mL) [8]. These differences are most probably related to the already mentioned different sensitivity of various commercial tests. A statistically significant elevation of IL-10 in patients with severe AP was obtained by other authors as well [14–16]. However, two other studies found significantly lower values of IL-10 in patients with severe AP [17, 18]. Authors speculated that an impaired immune response to inflammation could be a possible cause. It seems that a balance between pro- and anti-inflammatory cytokines is the key process in the course of AP and development of systemic complications. A reduced functional reserve of IL-10 and a higher IL-6/IL-10 ratio could lead to SAP and a worse prognosis. However, this is still a matter requiring further investigations. 

Results of sTNFr analysis, as another proinflammatory cytokine, were consistent with previously published results showing a significant elevation of serum concentrations in patients with SAP [19]. 

 The research included the analysis of two parameters that can be considered as valuable indicators of the course of disease; pancreatic elastase as a specific enzyme secreted by pancreatic acinar cells, and CRP as an acute phase protein, both increasingly produced and released in a state of acute inflammation. Elevated concentrations of E1 were measured in both groups of patients on admission, but without significant difference between the groups. However, we noticed a significant decline in concentration of E1 between the first and third day (*P* < 0.001), with a significant difference in values between the two groups patients (*P* = 0.001). Our results show that patients with a value of E1 below a cut-off value of 1.5 ng/mL measured on third day of admission could be considered potentially at low risk of development of systemic complications. 

A group of Australian authors in their examination of E1 concentrations in 29 patients with acute pancreatitis showed a sensitivity of 80%, and a sensitivity of 96% for a E1 cut-off value of 3.5 ng/mL measured on admission, with a sensitivity and specificity of 100% and 96%, respectively, on day three. The authors stated concurring limitations of ELISA tests, including lack of sensitivity due to questionable quality of used antibodies, and problematic reference range given by the manufacturer [20]. Similar limitations are present in all studies measuring serum concentrations of small amounts of antigens. Therefore, ELISA methods are not standardized and recommended for routine cytokine and elastase analyses, and should be used only for research purposes. Moreover, the implementation of the analysis in larger series, which includes sample collection and subsequent determination, makes these methods unsuitable for routine use. Development of homogenous immunochemical methods appropriate for automated and standardized determination of individual samples should increase the prognostic value and practical application of different biochemical parameters. 

As well as for E1 analysis, the concentrations of CRP differed significantly between day one and three (*P* < 0.001). Patients who developed systemic complications showed significantly higher levels of CRP (*P* = 0.002). ROC analysis showed and AUC of 0.69 for a cut-off value of 214 mg/L, with a rather low sensitivity and specificity, 54% and 84%, respectively. Gürleyik et al. showed similar results with sensitivity of 85%, and specificity of 74%, although for a cut-off value that was significantly different than in our study, 193 mg/L versus 214 mg/L, respectively [21]. Other authors also point out the value of CRP as a tool for AP severity assessment [22–24]. 

 We confirmed that CRP and elastase analyzed on the third day of admission, in addition to the evaluation of IL-6, IL-8, IL-10, and sTNFr on the first day, represent a valuable diagnostic tool in the assessment of severity and course of disease in patients with acute pancreatitis. Nevertheless, CRP is still the only recommended and standardized method for a fast and relatively inexpensive determination of severity of AP. Routine use of proinflammatory cytokines as predicting factors of severity of acute pancreatitis is still not feasible in most hospitals, due to high costs and inaccessibility of analytic methods. Therefore, development of new and more accessible laboratory equipment, as well as methods of analysis could help the clinicians in the early recognition of development of systemic complications and improve the management of severe acute pancreatitis.

## Figures and Tables

**Table 1:** Average values of biochemical parameters in patients with acute pancreatitis and the respective reference values according to manufacturers' recommendations.

Parameters	Median (95% CI)	Reference values
**IL-6 (1st day)**	**29 (18–46)**	**3.13–12.5 pg/mL**
IL-8 (1st day)	30 (23–39)	≤31.2 pg/mL
IL-10 (1st day)	7.4 (3.9–11.2)	≤7.8 pg/mL
sTNFr (1st day)	1698 (1419–1998)	749–1966 pg/mL
**CRP (1st day)**	**15.2 (12–27)**	**≤5 mg/L**
**CRP (3rd day)**	**103.6 (80–139)**	**≤5 mg/L**
**Elastase (1st day)**	**5.9 (4.3–7.5)**	**≤3.5 ng/mL**
Elastase (3rd day)	1.8 (1.6–2.2)	≤3.5 ng/mL

**Table 2:** Significant difference of average CRP and elastase values measured on the first and third day of admission in patients with acute pancreatitis.

Parameters	Median (95% CI)	*P* value
CRP (1st day) [mg/L]	15 (12–27)	*P* < 0.001
CRP (3rd day) [mg/L]	104 (80–139)	

Elastase (1st day) [ng/mL]	5.9 (4.3–7.5)	*P* < 0.001
Elastase (3rd day) [ng/mL]	1.8 (1.6–2.2)	

**Table 3:** Values of biochemical parameters measured in patients without systemic complications (*N* = 122) and patients who developed systemic complications (*N* = 28) and comparison of measured values. Bold printed are the indicators with statistically significant difference.

Parameters	Patients without systemic complications (*N* = 122)	Patients with systemic complications (*N* = 28)	*P* value
	Median (95% CI)	Median (95% CI)	
**IL-6 (1st day) [pg/mL]**	20 (16–34)	104 (51–139)	**0.001**
**IL-8 (1st day) [pg/mL]**	27 (19–34)	53 (33–98)	**0.012**
**IL-10 (1st day) [pg/mL]**	5.1 (2.6–8.9)	26 (8–58)	**0.010**
**sTNFr (1st day) [pg/mL]**	1520 (1220–1886)	2220 (1873–2722)	**0.004**
CRP (1st day) [mg/L]	14.4 (11.3–28.3)	15 (5.1–78.8)	0.954
**CRP (3rd day) [mg/L]**	85 (66.7–122)	216 (105–257)	**0.002**
Elastase (1st day) [ng/mL]	5.7 (4.2–7.5)	7.5 (3.6–10.9)	0.249
**Elastase (3rd day) [ng/mL]**	1.7 (1.4–1.9)	2.5 (2.1–5.1)	**0.001**

**Table 4:** ROC analysis for biochemical parameters of patients with acute pancreatitis. The criterion of classifying patients is the occurrence or absence of systemic complications of the disease. Bold printed are the indicators with statistically significant difference.

Parameters	AUC (95% CI)	*P* value	Cut-off	Sensitivity (%)	Specificit (%)	PPV (%)	NPV (%)
**IL-6 (1st day) [pg/mL]**	0.71 (0.63–0.78)	**<0.001**	37.9	82	65	35	94
**IL-8 (1st day) [pg/mL]**	0.66 (0.58–0.74)	**0.008**	42.5	68	67	32	90
**IL-10 (1st day) [pg/mL]**	0.65 (0.57–0.73)	**0.013**	7.2	75	56	28	91
**sTNFr (1st day) [pg/mL]**	0.69 (0.60–0.76)	**0.002**	1552	82	53	29	93
CRP (1st day)	0.51 (0.43–0.60)	0.842	85	32	86	35	85
**CRP (3rd day) (mg/L)**	0.69 (0.61–0.77)	**0.001**	214	54	84	44	89
Elastase (1st day) (ng/mL)	0.56 (0.48–0.64)	0.311	2.0	92	22	22	93
**Elastase (3rd day) (ng/mL)**	0.70 (0.62–0.77)	**<0.001**	1.5	93	43	27	96

## References

[1] Wu BU, Johannes RS, Sun X, Tabak Y, Conwell DL, Banks PA (2008). The early prediction of mortality in acute pancreatitis: a large population-based study. *Gut*.

[2] Frossard JL, Steer ML, Pastor CM (2008). Acute pancreatitis. *The Lancet*.

[3] Al-Bahrani AZ, Ammori BJ (2005). Clinical laboratory assessment of acute pancreatitis. *Clinica Chimica Acta*.

[4] Matull WR, Pereira SP, O’Donohue JW (2006). Biochemical markers of acute pancreatitis. *Journal of Clinical Pathology*.

[5] Bollen TL, van Santvoort HC, Besselink MG (2008). The Atlanta Classification of acute pancreatitis revisited. *British Journal of Surgery*.

[6] Bradley EL (1993). A clinically based classification system for acute pancreatitis: summary of the International Symposium on Acute Pancreatitis, Atlanta, Ga, September 11 through 13, 1992. *Archives of Surgery*.

[7] Dominguez-Munoz J (2007). *Clinical Pancreatology for Practising Gastroenterologists and Surgeons*.

[8] Štimac D, Fišić E, Milić S, Bilić-Zulle L, Perić R (2006). Prognostic values of IL-6, IL-8, and IL-10 in acute pancreatitis. *Journal of Clinical Gastroenterology*.

[9] Pezzilli R, Mokselli-Labate AM, Mintero R, Barakat B, Fiocchi M, Cappelletti O (1999). Simultaneous serum assays of lipase and interleukin-6 for early diagnosis and prognosis of acute pancreatitis. *Clinical Chemistry*.

[10] Jiang CF, Shiau YC, Ng KW, Tan SW (2004). Serum interleukin-6, tumor necrosis factor *α* and C-reactive protein in early prediction of severity of acute pancreatitis. *Journal of the Chinese Medical Association*.

[11] Chen CC, Wang SS, Lu RH, Lu CC, Chang FY, Lee SD (2003). Early changes of serum proinflammatory and anti-inflammatory cytokines after endoscopic retrograde cholangiopancreatography. *Pancreas*.

[12] Berney T, Gasche Y, Robert J (1999). Serum profiles of interleukin-6, interleukin-8, and interleukin-10 in patients with severe and mild acute pancreatitis. *Pancreas*.

[13] Pooran N, Indaram A, Singh P, Bank S (2003). Cytokines (IL-6, IL-8, TNF): early and reliable predictors of severe acute pancreatitis. *Journal of Clinical Gastroenterology*.

[14] Mayer J, Rau B, Gansauge F, Beger HG (2000). Inflammatory mediators in human acute pancreatitis: clinical and pathophysiological implications. *Gut*.

[15] Ohmoto K, Yamamoto S (2005). Serum interleukin-6 and interleukin-10 in patients with acute pancreatitis: clinical implications. *Hepato-Gastroenterology*.

[16] Simovic MO, Bonham MJD, Abu-Zidan FM, Windsor JA (1999). Anti-inflammatory cytokine response and clinical outcome in acute pancreatitis. *Critical Care Medicine*.

[17] Han XC, Zhang YC, Wang Y, Jia MK (2003). Clinical evaluation of serum interleukin 10 in patients with acute pancreatitis. *Hepatobiliary and Pancreatic Diseases International*.

[18] Pezzilli R, Billi P, Miniero R, Barakat B (1997). Serum interleukin-10 in human acute pancreatitis. *Digestive Diseases and Sciences*.

[19] Hirota M, Nozawa F, Okabe A (2000). Relationship between plasma cytokine concentration and multiple organ failure in patients with acute pancreatitis. *Pancreas*.

[20] Wilson RB, Warusavitarne J, Crameri DM, Alvaro F, Davies DJ, Merrett N (2005). Serum elastase in the diagnosis of acute pancreatitis: a prospective study. *ANZ Journal of Surgery*.

[21] Gürleyik G, Emir S, Kiliçoglu G, Arman A, Saglam A (2005). Computed tomography severity index, APACHE II score, and serum CRP concentration for predicting the severity of acute pancreatitis. *Journal of the Pancreas*.

[22] Imamura T, Tanaka S, Yoshida H (2002). Significance of measurement of high-sensitivity C-reactive protein in acute pancreatitis. *Journal of Gastroenterology*.

[23] Triantopoulou C, Lytras D, Maniatis P (2007). Computed tomography versus acute physiology and chronic health evaluation II score in predicting severity of acute pancreatitis: a prospective, comparative study with statistical evaluation. *Pancreas*.

[24] Triester SL, Kowdley KV (2002). Pancreatic and biliary disease. Prognostic factors in acute pancreatitis. *Journal of Clinical Gastroenterology*.

